# Detoxification and benefits on acute heart failure in mice- of fuziline using glycyrrhetinic acid: an integrated biochemical analysis

**DOI:** 10.3389/fphar.2024.1419663

**Published:** 2024-10-31

**Authors:** Nianwei Chang, Chunyu Hou, Yue Zhai, Wenying Zhang, Zengmei Hu, Xiaoying Wang

**Affiliations:** ^1^ School of Chinese Materia Medica, Tianjin University of Traditional Chinese Medicine, Tianjin, China; ^2^ Pharmacy Dept, Ma’anshan Hospital of Traditional Chinese Medicine, Ma’anshan, Anhui, China

**Keywords:** fuziline, glycyrrhetinic acid, heart failure, synergetic compatibility, MDH2, calcium signaling

## Abstract

**Introduction:**

Aconiti Lateralis Radix Praeparata (lateral roots of *Aconitum carmichaelii* Debeaux, Fuzi), is commonly used to treat various cardiovascular diseases, particularly heart failure. However, its strong cardiotoxicity limits its clinical applicability. Glycyrrhizae radix et rhizoma, (the root of *Glycyrrhiza uralensis* Fisch., Gancao), is known to synergistically increase the cardiotonic effects of Fuzi and alleviate the myocardial injury caused by Fuzi to some extent. However, the detailed mechanism via which the combination of Fuzi and Gancao reduces toxicity and increases or preserves the efficacy of Fuzi requires further investigation.

**Methods:**

Oxidative stress injury models in H9C2 cells and mice with acute heart failure were established to evaluate the optimal synergistic protective concentration of Fuziline and Glycyrrhetinic acid (GA). A GA probe was then synthesized and used for target fishing using chemical and biological methods. Finally, the target and its function were verified using fluorescence co-localization, Western blotting, protein interaction analysis, molecular docking, and calcium ion imaging.

**Results:**

The best pharmacodynamic potential was achieved with a 1:1 or 2:1 ratio of Fuziline and GA concentrations. At these ratios, they regulated the protein levels of the downstream players of the Ca^2+^ signaling pathway via MDH2 and CALR, thereby balancing Ca^2+^ homeostasis in the myocardial tissue and mitigating the effects of heart failure.

**Conclusion:**

This study aimed to investigate the compatibility of Fuziline and GA, the active metabolites of a traditional Chinese medicine (TCM) pair, in exerting their cardiac effects, identify the direct biological targets and verify the mechanism of compatibility.

## 1 Introduction

Heart failure (HF) is defined as the inability of the heart to supply blood and oxygen to the surrounding tissues, which adversely affects their metabolic needs. A common “epidemic” in modern society, sudden cardiac death or multiple organ failure caused by chronic systemic hypoperfusion is the main cause of death in patients with HF (Lane et al., 2005). Despite the rapid development of treatment strategies, the prognosis of HF remains poor. The high mortality, incidence rate, and hospitalization demands of patients continue to exert pressure on clinical and public health systems. Activation of the inflammatory cytokine system, oxidative stress, and imbalance in Ca^2+^ homeostasis promote HF, the terminal stage of cardiovascular diseases (Rogers and Bush, 2015). In the pathological state, the surge in reactive oxygen species (ROS) levels in cardiomyocytes exceeds the buffering capacity of the antioxidant system. Activation of Ca^2+^/calmodulin-dependent protein kinase II (CAMKII) also causes Ca^2+^ leakage in the sarcoplasmic reticulum, resulting in myocardial damage (Reyes Gaido et al., 2023).

Aconiti Lateralis Radix Praeparata (lateral roots of *Aconitum carmichaelii* Debeaux, Fuzi), is a traditional Chinese medicine (TCM) widely used for treating cardiovascular diseases. It is effective against conditions such as HF, hypotension, coronary heart disease, and shock caused by myocardial infarction (Xing et al., 2022). However, Fuzi is highly toxic and has been classified as an inferior product in the Shen Nong Ben Cao Jing (The Divine Husbandman’s Classic of the Materia Medica) that is “not to be used for non-critical illnesses”, thereby restricting its clinical applicability. Fuziline, the main metabolite of Fuzi absorbed by the blood, possesses cardiotonic, anti-myocardial, ischemic, anti-shock, and anti-arrhythmic properties. Fuziline can inhibit the isoproterenol-induced apoptosis of myocardial cells, improve the viability of myocardial cells, regulate the PERK/EIF2-α/ATF4/Chop pathway, inhibit ROS-induced endoplasmic reticulum stress, maintain the stability of mitochondrial membrane potential, and inhibit cytochrome C release to the cytoplasm (Fan et al., 2020).

Glycyrrhizae radix et rhizoma, (the root of *Glycyrrhiza uralensis* Fisch., Gancao), a classic compatible partner of Fuzi, not only retain the original cardiotonic and analgesic activities of Fuzi, but also reduce myocardial damage caused by Fuzi and affect the metabolism of toxic metabolites to achieve detoxification (Ke et al., 2015). Glycyrrhetinic acid (GA) can restore Fuzi-induced decline in myocardial Na^+^-K^+^-ATPase activity, promote Na^+^-K^+^ exchange, inhibit Na^+^-Ca^2+^ exchange, alleviate the Ca^2+^ overload, reduce the activity of calmodulin/CAMKII, and antagonize aconitine-induced arrhythmia in rats.(Zhang et al., 2017). Previously, we showed that Fuziline, a Ca^2+^ and β_2_-AR agonist, exhibited significant cardiotonic and anti-myocardial ischemic effects and also could lead to arrhythmia. GA can inhibit the Fuziline-induced increase in Ca^2+^ levels while retaining the activation of the β_2_ adrenergic receptor (β_2_-AR), thus eliminating the side effects of GA without influencing the cardiotonic effect (Chang et al., 2020). However, the specific mechanism by which the Fuzi and GA combination synergistically reduces toxicity and increases/preserves their beneficial effect remains unclear, and their clinical applicability remains unestablished.

Hence, in this study, we aimed to identify the direct biological target of Fuziline and GA and elucidate their mechanisms of action. To achieve this, we first analyzed the optimal ratio for synergistic protective effect of Fuziline and GA on oxidatively damaged cardiomyocytes and evaluated its mitigating effect in mice with HF. Subsequently, small-molecule probes were used to identify the target proteins, and various biochemical experiments were performed to assess the impact of potential targets and the effect of Fuziline and GA.

## 2 Methods and materials

### 2.1 Reagents and materials

Fuziline (CAT #A0793, purity ≥98%) and GA (CAT #A0038, purity ≥98%) were purchased from Must Bio-Technology Co., Ltd. (Chengdu, China). The GA probe was supplied by Wuxi AppTec. Co., Ltd. (Tianjin, China).

### 2.2 Animals

All experimental protocols were performed in accordance with the Guide for the Care and Use of Laboratory Animals and approved by the Animal Ethics Review Committee of Tianjin University of Traditional Chinese Medicine (TCM-LAEC2021136). C57BL/6JNifdc mice (male, 20–25 g) were purchased from the Beijing Weitong Lihua Animal Experiment Center (SCXK2018−0004, Beijing, China). The mice were maintained under a 12 h automatic light/dark cycle in a standard animal room at 20–25°C. The mice had *ad libitum* access to water and food.

### 2.3 Detection of acute heart failure (AHF) in mice

Male mice were randomly assigned to 11 groups (n = 6 in each group). Except for the control group, other groups were administered the following treatments 24 h after establishing the AHF model using doxorubicin (Dox) (intraperitoneal injection [i.p.] 15 mg/kg): (1) control group (Con, i.p. saline), (2) model group (M, i.p. saline), (3–6) Fuziline groups (F, i.p. 0.08, 0.16, 0.32, and 0.64 mg/kg), (7–10) combination groups (Fuziline with GA (FG), i.p. concentration 1:1), and (11) positive group (dexrazoxane, DEX, i.p. 150 mg/kg). After administration for 7 days, the mice were examined using a small animal real-time ultrasound imaging system (Vevo 2,100, Visual Sonics, Canada), and the indices of heart function, such as left ventricular ejection fractions, left ventricular fractional shortening, and cardiac output, were determined. Subsequently, serum samples were collected, and the degree of HF was evaluated using the lactate dehydrogenase (LDH), cardiac troponin (CTnT), creatine kinase (CK), and calcineurin (CAN) enzyme-linked immunosorbent assay kits. Heart tissues were collected for immunofluorescence analysis and morphological observation using hematoxylin and eosin (H&E) staining.

### 2.4 Cell culture

The H9C2 rat myocardium-derived cell line (H9C2) was obtained from the American Type Culture Collection (Manassas, VA, USA). H9C2 cells were cultured in Dulbecco’s modified Eagle’s medium supplemented with 10% fetal bovine serum, 100 U/mL penicillin, and 0.1 mg/mL streptomycin in a 37°C humidified incubator in an atmosphere of 5% CO_2_.

### 2.5 Analysis of compatibility concentration

H9C2 cells were stimulated with 200 μM H_2_O_2_ and cultured in a 96-hole board for 2 h. According to the rule of the chessboard method, Fuziline and GA were added into each hole to achieve final concentrations of 0.8, 0.4, 0.2, 0.1, 0.05, and 0 μM. After incubation for 10 h, the 3-[4,5-dimethylthiazol-2-yl]-2,5 diphenyl tetrazolium bromide (MTT) and LDH assays were performed to determine the effect of varying Fuziline and GA concentrations on H9C2 cells.

### 2.6 Target capture

The mice were divided into four groups, including the control group, which were stimulated with GA, Fuziline with the GA probe (1:1), and GA probe i.p. for 3 days. One hour after the last day of administration, protein lysates were extracted from the heart tissues for target capture.

The cells were divided into four groups. After 8–10 h of starvation, fresh culture medium was added to the control group and drugs were added to the other groups. Cellular proteins were extracted after 10 h of incubation.

Azide-modified magnetic microspheres (MMs) were prepared as described in Figure 3A. The MMs were added to the protein lysates along with CuSO_4_ (1 mM) and Tris (2-carboxyethyl) phosphine hydrochloride (TCEP) (1 mM) and incubated for 12 h at 4°C. The MMs were collected by magnetic separation and rinsed with precooled phosphate-buffered saline. The bound proteins were reduced using 300 μL dithiothreitol (100 mM) at 4°C for 30 min, and then the supernatants were collected for 15% sodium dodecyl sulfate-polyacrylamide gel electrophoresis (SDS-PAGE). Finally, the protein bands were digested using trypsin and the resulting peptides were analyzed using liquid chromatography-tandem mass spectrometry (LC-MS/MS) to identify the proteins captured by GA, GA probe, and Fuziline with the GA probe. The LC-MS/MS analysis was performed by Beijing Protein Innovation Co., Ltd. (Beijing, China).

### 2.7 Co-localizationAssay

H9C2 cells were treated with the GA probe and GA probes with Fuziline for 6 h and then fixed using 4% paraformaldehyde. The fixed cells were incubated with azido-coumarin tracer in click buffer (1 mM CuSO4, 1 mM TCEP, and 0.1 mM TBTA in 10% dimethyl sulfoxide) for 2 h at 37°C. The cells were then incubated with the malate dehydrogenase 2 (MDH2) antibody (1:500) at 4°C for 12 h and with Alexa Fluor 594-conjugated goat anti-mouse antibody (1:1,000) for 30 min at 37°C. Fluorescence imaging was performed using a laser scanning confocal microscope (Leica SP8, Carl Zeiss, Oberkochen, Germany). The GA probe was detected at 488/516 nm (Ex/Em) and MDH2 was detected at 594/617 nm (Ex/Em).

### 2.8 Target protein prediction

The structures of Fuziline and its potential targets were constructed and minimized using the Molecular Operating Environment software package (Chemical Computing Group, Inc.). Auto Dock version 4.2 (Olson Laboratory, La Jolla, CA) was used to simulate ligand-receptor binding using a hybrid Lamarckian genetic algorithm. The step size parameters for the quaternion and torsion were set to 30. Thirty independent experiments were performed for each metabolite. Default values were used for all other parameters. The free energy of binding was calculated from the contributions of hydrophobic, ionic, and hydrogen bonds, and van der Waals interactions between proteins and ligands.

### 2.9 Detection of damage in H9C2 cells

An H9C2 cell injury model was established using Dox. The experimental cells were divided into the following 10 groups, (1) control group (Con), (2) model group (M, Dox), (3–6) Fuziline groups (0.01, 0.1, 1, and 10 μM), and (7–10) combination groups (Fuziline with GA, concentration 1:1). After the cells were stimulated with 4 μM Dox for 2 h, all groups, except for the control group, were administered Fuzuline or GA and incubated for 22 h. Calcium fluorescence intensity, F-actin staining, and immunofluorescence were detected in the H9C2 cells. The cell supernatants were collected to measure ryanodine receptor 2 (RyR2) expression.

### 2.10 Protein thermal shift and drug affinity responsive target stability (DARTS) assay

For performing a thermal shift assay in heart tissue samples, the proteins were exposed to 10^−5^ mol/L metabolites and incubated overnight at 4°C. Subsequently, the proteins were centrifuged at 12,000 rpm for 10 min at 4°C and the supernatants were transferred to 1.5 mL microfuge tubes. After preparation of the lysates, aliquots of the supernatants were heated individually on a Thermomixer Compact at different temperatures for 5 min and then cooled to 24°C. After centrifugation at 12,000 rpm for 10 min at 4°C, the supernatants were collected for Western blotting.

For DARTS analysis, proteins were diluted with varying concentrations of streptomycin in TNC buffer to achieve enzyme and protein concentration ratios of 1:200, 1:400, 1:600, 1:800, and 1:1,000. After 3 min of enzymatic hydrolysis at room temperature, the proteins were heated in a metal bath for Western blotting.

### 2.11 Western blot analysis

Proteins were separated using SDS-PAGE and transferred to polyvinylidene fluoride membranes. The membranes were blocked with 5% nonfat dry milk at 24°C–26°C for 1 h and then incubated with primary antibodies against calreticulin (CALR), aspartate beta-hydroxylase (ASPH), MDH2, myocyte enhancer factor 2C (MEF2C), calsequestrin 2 (CASQ2), and glyceraldehyde 3-phosphate dehydrogenase (CST, MA, USA) overnight at 4 °C. The membranes were washed using Tris-buffered saline with 0.1% Tween 20 for 30 min, incubated with a secondary antibody (CST, MA, USA) at 24°C–26°C for 3 h, and washed again. The membranes were incubated with chemiluminescent horseradish peroxidase substrates and exposed to enhanced chemiluminescence and Western blot detection reagents (GE Healthcare, Buckinghamshire, UK).

### 2.12 Protein-protein interaction analysis

Purified MDH2 (20 mg/mL) was fixed onto a SMAP chip and Fuziline was used as the mobile phase to compete with the protein for binding and dissociation. The flow rate was set to 30 mL/min and continuously monitored for 700 s. Data were collected to calculate the dissociation constant.

### 2.13 Statistical analysis

All data were expressed as means ± standard deviation, where significant differences between two and multiple groups were analyzed using Student’s t-test and analysis of variance, respectively, of Graph Pad Prism 7.0. Statistical significance was set at *p* < 0.05.

## 3 Results

### 3.1 Effect of different concentration ratios of fuziline and GA on H9C2 cells

**FIGURE 1:**
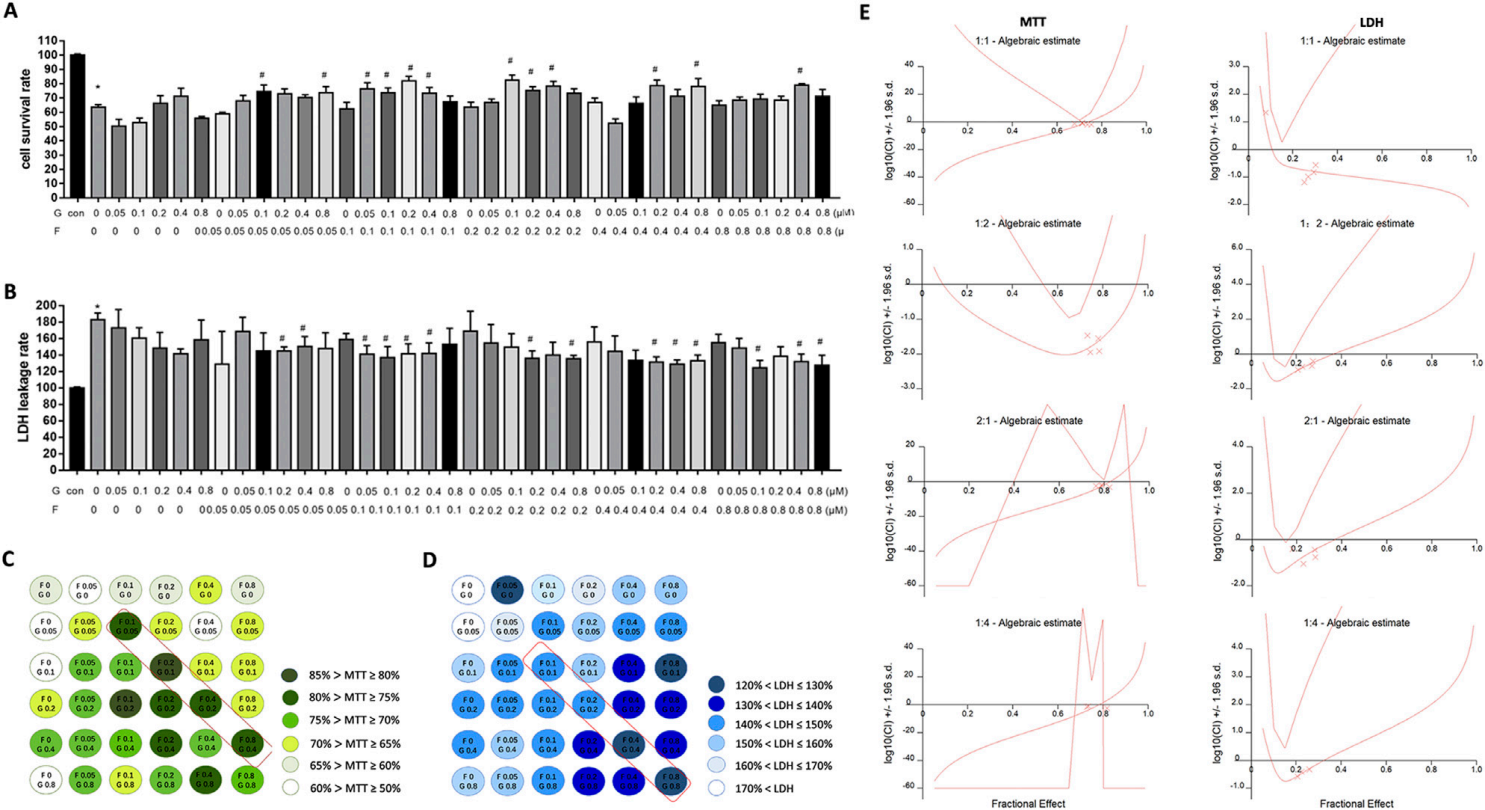
The effect of different proportions compatibility of Fuziline and GA on cells. **(A)** H9C2 cells survival rate. **(B)** H9C2 cells LDH leakage rate. **(C)** Visibility graph in H9C2 cell survival rate with chessboard style compatibility. **(D)** Visibility graph in H9C2 cell LDH leakage rate with chessboard style compatibility. **(E)** Synergistic index diagram of different proportions of Fuziline and GA.

To determine the optimal compatibility ratio of Fuziline and GA, an oxidative stress injury model of H9C2 cells was established using H_2_O_2_. The histograms and visual graphs (Figures 1A, C) revealed that cell survival rate was the highest when 2:1 ratio of Fuziline: GA was used. Analysis of LDH leakage rate (Figures 1B, D) revealed that the leakage rate of LDH in the cell supernatant was the lowest when the Fuziline: GA ratio was 1:1.

To further determine the optimal ratio, the synergetic index (CI) values for different ratios were calculated using the CalcuSyn software and the combined effect of Fuziline and GA was evaluated according to the Chou-Talalay medium effect principle. We observed that the CI values of MTT and LDH were less than one when the Fuziline concentration ranged from 0.1 μM to 0.8 μM and when Fuziline: GA was applied in a 1:1 or 2:1 ratio, indicating the synergistic effect of Fuziline and GA at this concentration ratio (Figure 1E).

### 3.2 Pharmacodynamic evaluation of the effect of fuziline and GA’s compatibility on mice with AHF

**FIGURE 2:**
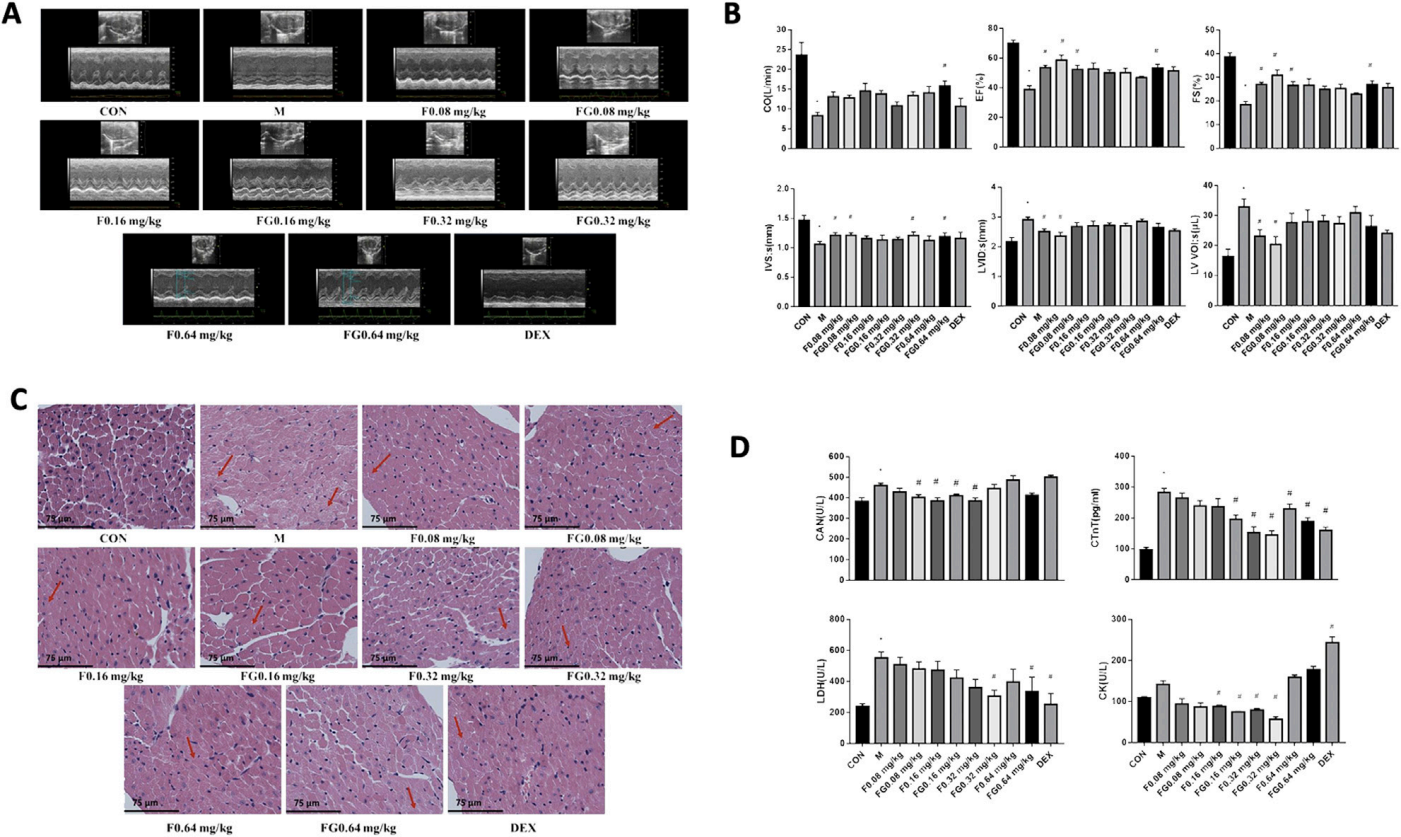
Effects of Fuziline and GA on Mice with AHF. **(A)** Ultrasound M-Mode Long Axis Section. **(B)** Effects of different administration groups on cardiac function in mice with acute heart failure. **(C)** Morphology of the heart of mice in different administration groups. **(D)** Content changes of CAN, CTnT, LDH, and CK in different administration groups (n = 6).

We determined that the 1:1 combination of Fuziline and GA was optimal and validated its effect on mice with AHF. As shown in Figure 2A, compared to the control group, the model group showed obvious thinning of the ventricular wall, volume expansion, slower wall movement, and lower contractility. Compared to the model group, the administration group showed some improvement. After 7 days of continuous administration, M-mode was used to detect the long-axis section and evaluate the indicators of cardiac function (Figure 2B). Cardiac output (CO), ejection fraction (EF), fraction shorting (FS), and interventricular septum (IVS) in the model group was significantly lower than those in the control group (*p* < 0.05), whereas left ventricular systolic inner diameter (LVID; s) and left ventricular systolic volume (LV VOL; s) were significantly higher (*p* < 0.05). Compared with the model group, the FG 0.64 mg/kg group showed significantly decreased CO (*p* < 0.05), and the F 0.08 mg/kg, FG 0.08 mg/kg, F 0.16 mg/kg, and FG 0.64 mg/kg groups showed significantly decreased EF and short axis shortening rate (*p* < 0.05). The F 0.08 mg/kg, FG 0.08 mg/kg, F 0.32 mg/kg, and FG 0.64 mg/kg groups showed significantly reduced left ventricular posterior wall thickness at the end of contraction (*p* < 0.05). The F 0.08 mg/kg and FG 0.08 mg/kg groups showed a tendency of reduction in the left ventricular systolic diameter (*p* < 0.05), whereas the F 0.08 mg/kg and FG 0.08 mg/kg groups showed significantly reduced left ventricular systolic volume (*p* < 0.05).

The H&E stained heart sections of mice in the control group did not show any remarkable change in the myocardial layer (Figure 2C). The myocardial cells were arranged in an orderly fashion and the myocardial gap was normal. Analysis of the model group revealed light-colored myocardial cell cytoplasm with loose cell morphology, myocardial edema, and inflammatory cell infiltration. The administration groups showed improvement in myocardial cell edema and inflammatory cell infiltration to varying degrees, exhibiting a certain myocardial protective effect.

The mitigation effect of the Fuziline and GA compatibility groups on mice with HF was evaluated based on changes in the serum levels of the biomarkers, CAN, LDH, CK, and CTnT (Figure 2D). The results showed that the CAN levels in the FG 0.08, F 0.16, FG 0.16, and F 0.32 mg/kg groups were significantly lower than those in the model group (*p* < 0.05). The CTnT levels in the FG 0.16, F 0.32, FG 0.32, F 0.64, FG 0.64 mg/kg, and DEX groups decreased significantly (*p* < 0.05), the LDH levels in the FG 0.32 mg/kg, FG 0.64 mg/kg, and DEX groups decreased significantly (*p* < 0.05), the CK levels in the model group showed an upward trend, and the serum CK levels in the F 0.16, FG 0.16, F 0.32, and FG 0.32 mg/kg groups decreased significantly (*p* < 0.05), while the CK levels in the F 0.64 mg/kg, FG 0.64 mg/kg, and DEX groups increased significantly (*p* < 0.05). These observations suggested that the Fuziline and compatibility groups improved AHF in mice to a certain extent.

### 3.3 Direct target fishing and verification of the compatibility of GA with fuziline

**FIGURE 3:**
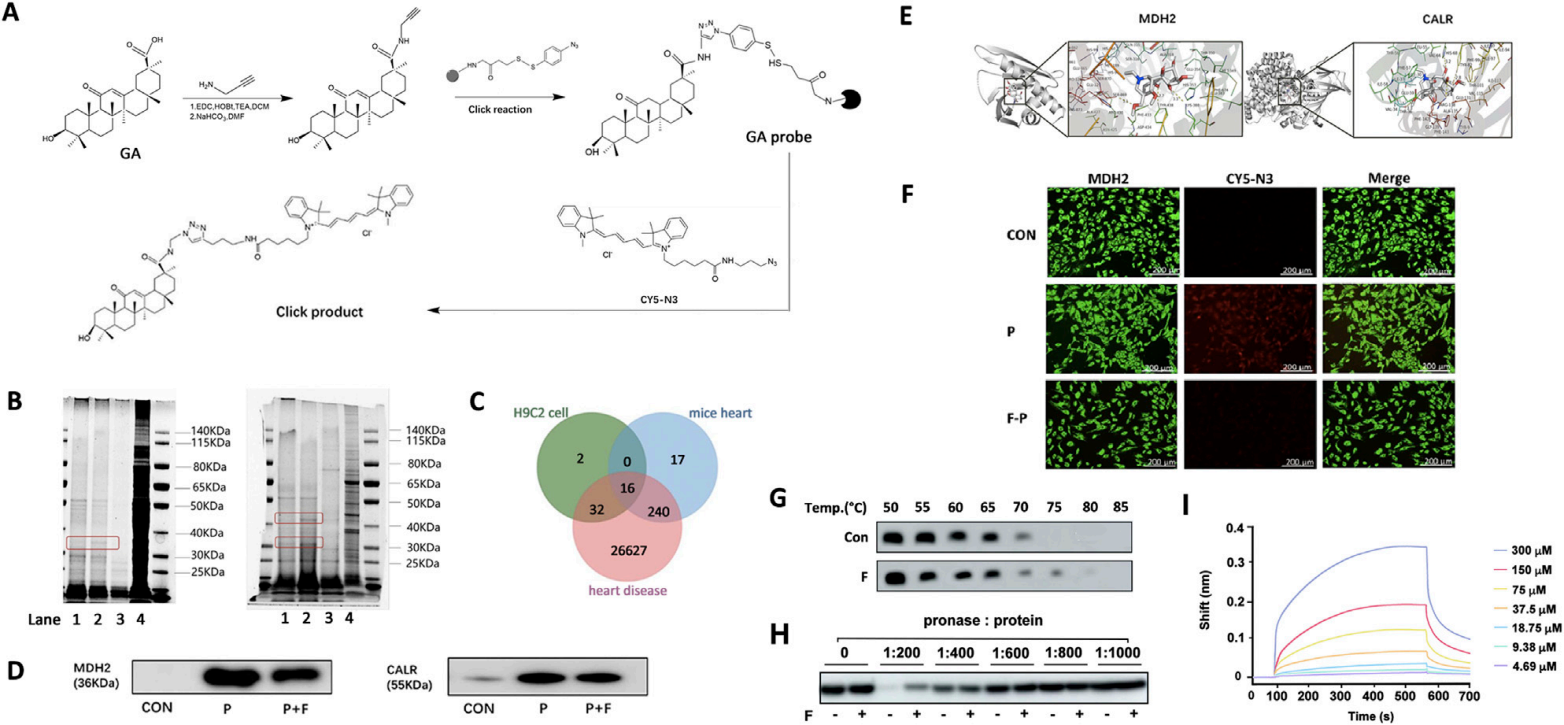
Direct target fishing and verification. **(A)** Preparation of GA functionalized magnetic microspheres. **(B)** Evaluation of the protein capture ability of GA probe by SDS-PAGE. **(C)** Schematic diagram of the overlapping targets of GA probe and Fuziline. **(D)** Western blot validation analysis of target captured by GA probe. **(E)** Molecular docking verification of Fuziline with MDH2 and CALR. **(F)** Fluorescence colocalization analysis of GA probe (red) and MDH2 protein (green). **(G)** The thermal stabilization of MDH2 by Fuziline. **(H)** The DARTS assay of MDH2 by Fuziline. **(I)** Bio-layer interferometry.

To identify the biological target proteins before and after testing the compatibility of the two metabolites, a GA probe was synthesized for the click chemical reaction (Figure 3A). Several proteins were effectively captured in the cells and heart tissues of the compatibility groups. Protein bands between 30 and 50 kDa were observed at different depths. This may be due to the competitive binding of Fuziline to the GA probe (Figure 3B).

Q Exactive LC-MS/MS was used for protein identification. Analysis of the LC-MS spectrum revealed 50 proteins in the cells and 240 proteins in the heart tissue (Figure 3C), among which 16 proteins were related to heart disease-related targets in the GeneCard database. Further analysis led to the identification of the following four proteins related closely to Ca^2+^ regulation: MDH2, CALR, receptor for activated C kinase 1 (RACK1), and CRK proto-oncogene adaptor protein (CRK). Among them, MDH2 and CALR showed the highest comprehensive scores in the cells and heart tissue; hence we performed a follow-up experimental verification for MDH2 and CALR. Western blotting revealed that Fuziline could competitively bind to MDH2, thus reducing the amount of protein captured by the GA probe, however, the CALR concentration did not decrease significantly after adding Fuziline (Figure 3D). Therefore, MDH2 was considered a potential target of Fuziline.

Furthermore, the AutoDockTools 1.5.6 software was used to verify molecular docking between MDH2, CALR, and Fuziline (Figure 3E). Fuziline could associate with Ser869, Tyr438, and Lys380 sites of MDH2 via hydrogen bonds with a binding energy of −7.22 kcal/mol. Fuziline could associate with His68, Tyr82, Arg138, and Thr36 of CALR via hydrogen bonds with a binding energy of −8.01 kcal/mol.

A fluorescence colocalization experiment was performed to verify the binding of GA and Fuziline to MDH2 in H9C2 cells. Compared to that in the control group, the GA probe group showed a strong red fluorescence (Figure 3F), which decreased with the addition of Fuziline. This indicated that Fuziline and the GA probe were bound to the same target and that the amount of GA probe binding and fluorescence intensity decreased due to the competitive binding of Fuziline with MDH2.

In this study, Fuziline exerted its toxic effects by targeting MDH2. Therefore, we conducted protein-binding assays using the protein lysates of the cardiac tissue. Compared to that observed in the control group, the thermal stability of MDH2 increased upon treatment with Fuziline at 75°C, and Fuziline protected MDH2 from hydrolysis (Figures 3G, H). Using Gator, the binding constant between Fuziline and purified MDH2 was found to be 17.08 μM (Figure 3I), which was further confirmed by the interaction between Fuziline and MDH2.

### 3.4 Regulation of dox-induced injuries by the compatibility of fuziline and GA

**FIGURE 4:**
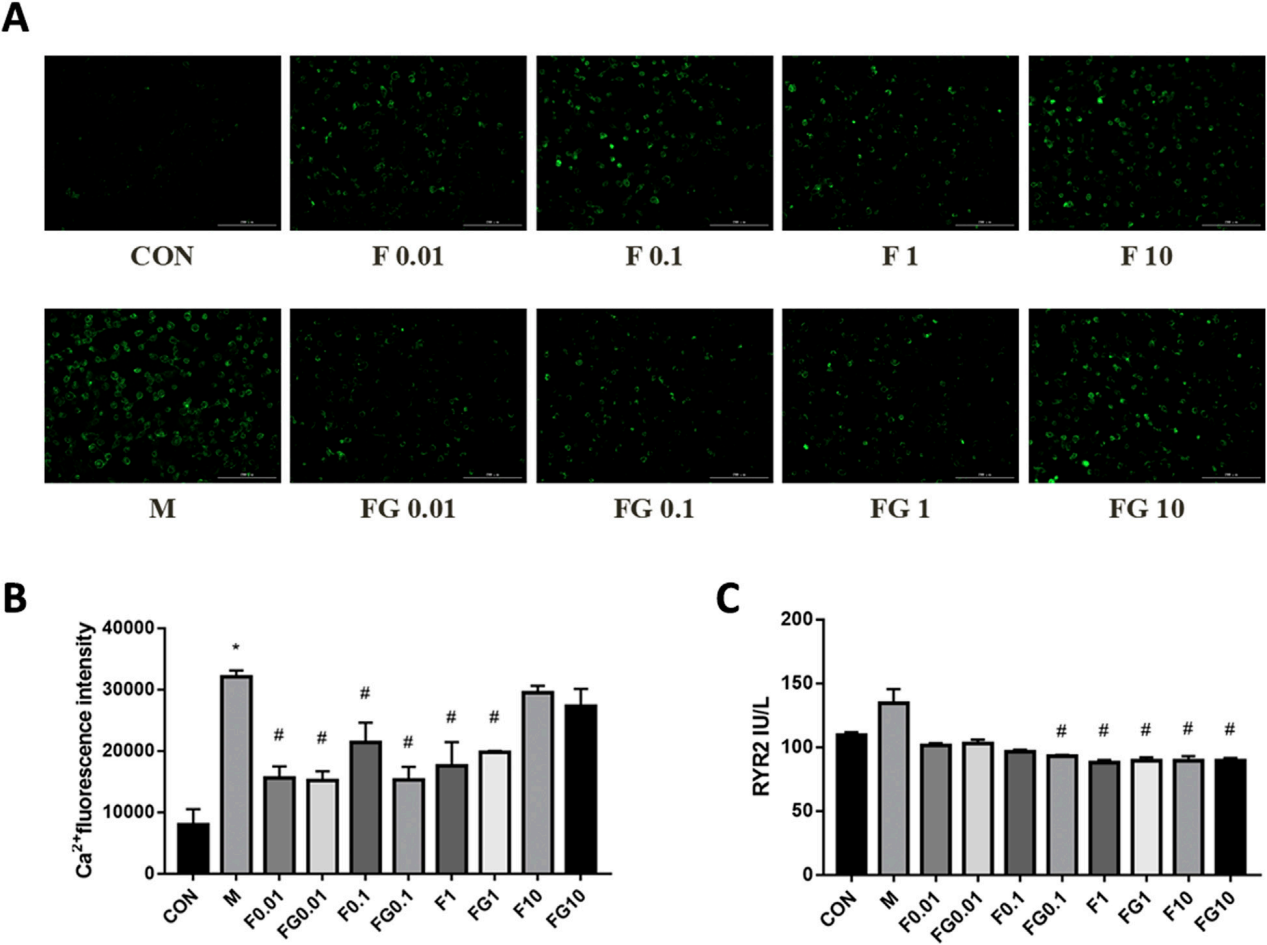
Effects of fuziline and GA on cellular damage caused by Dox. **(A)** Changes in calcium ion fluorescence intensity after 22 h of different administration concentrations. **(B)** Quantitative map of changes in intracellular calcium ion concentration. **(C)** Changes in the expression of ryanodine receptors after 22 h of different administration concentrations.

A Dox injury model was developed to investigate the effects of Fuziline and GA *in vitro*. Fuziline mitigated arrhythmia or even HF by increasing the Ca^2+^ content, which could be alleviated by GA. Therefore, we investigated the regulatory effects of Fuziline and GA compatibility on Ca^2+^ levels in injured H9C2 cells. The results of Ca^2+^ imaging showed that fluorescence intensities decreased significantly in the Fuziline and compatibility groups compared with that in of the model group (*p* < 0.05) when the Fuziline concentrations were 0.01, 0.1 and 1 μM. However, in the presence of 10 μM Fuziline, the Ca^2+^ fluorescence intensity in the treatment group did not differ significantly from that in the model group (Figures 4A, B), indicating that this Fuziline concentration could not inhibit the Ca^2+^ overload in H9C2 cells. We found that the Dox-induced increase in RyR2 expression was significantly alleviated in the Fuziline (1 μM and 10 μM) and compatibility groups (0.1, 1, and 10 μM) (*p* < 0.05) (Figure 4C).

### 3.5 Fuziline and GA mitigated HF by regulating the calcium signaling pathway

**FIGURE 5:**
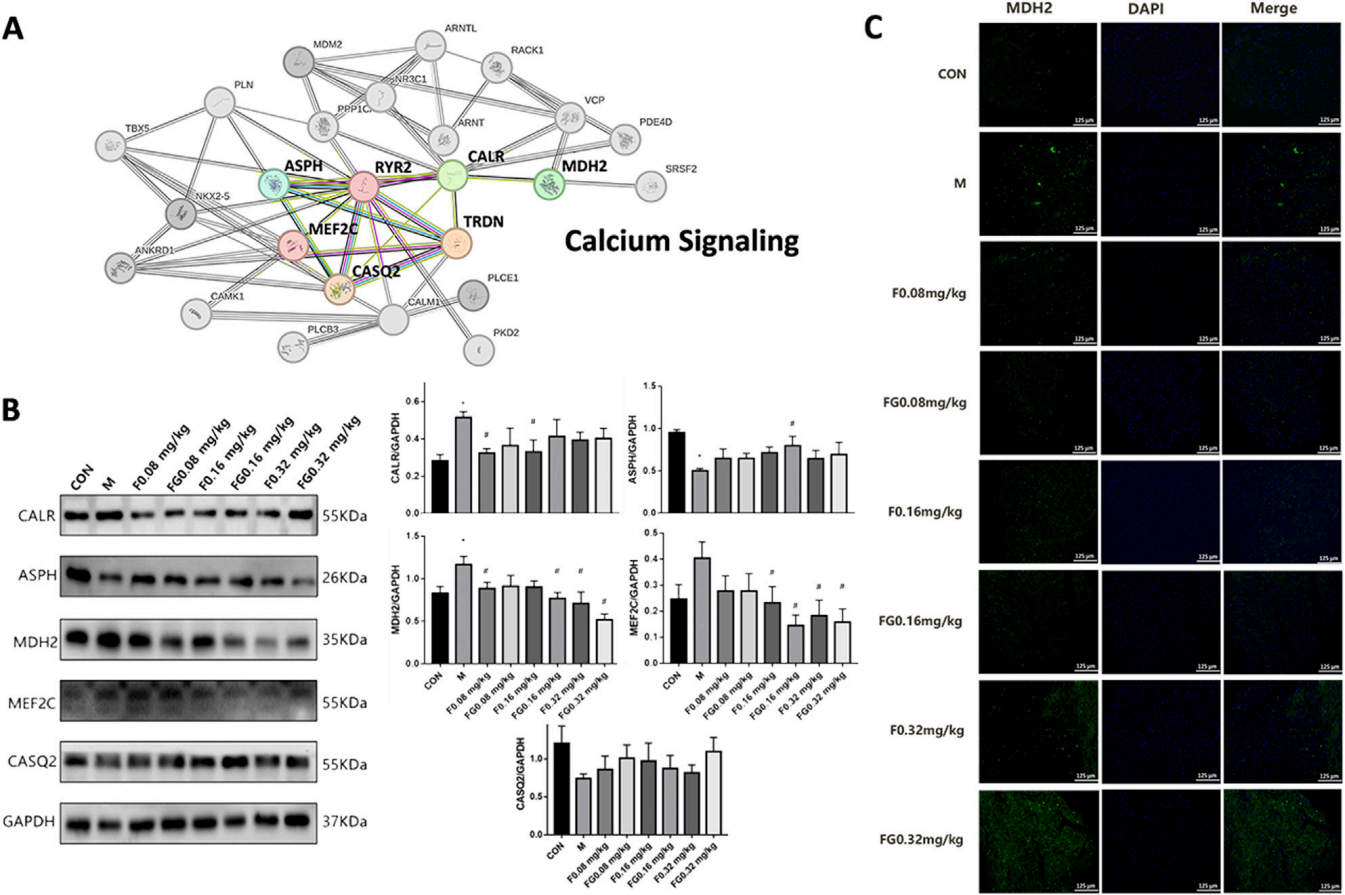
Regulation of Ca^2+^ signaling pathway by aconite and GA. **(A)** Interaction network diagram between calcium-related proteins. **(B)** Expression of CALR, ASPH, MDH2, MEF2C and CASQ2 proteins in myocardial tissue. **(C)** Immunofluorescence of MDH2 expression in the heart of the administration group (n = 6).

To determine the mechanism via which the compatibility of Fuziline and GA regulated Ca^2+^ levels, Ingenuity Pathway Analysis (IPA) was used to analyze and integrate the Ca^2+^ -related proteins. Figure 5A shows that MDH2 interacted with CALR and co-regulated the downstream proteins involved in the Ca^2+^ signaling pathway, namely, MEF2C, RyR2, CASQ2, triadic (TRDN), and ASPH. MEF2C protein level increased, whereas those of CASQ2 and ASPH decreased when CALR was upregulated.

Next, we conducted Western blot experiments on mice with AHF. The expression of CALR and MDH2 in the model group increased significantly (*p* < 0.05), whereas that of ASPH decreased significantly (*p* < 0.05) (Figure 5B). Compared with the model group, the F 0.08 mg/kg and F 0.16 mg/kg groups showed significantly decreased expression of CALR (*p* < 0.05); the F 0.16 mg/kg, FG 0.16 mg/kg, F 0.32 mg/kg, and FG 0.32 mg/kg groups showed significantly decreased MEF2C protein expression (*p* < 0.05); the F 0.08, FG 0.16, F 0.32, and FG 0.32 mg/kg groups showed significantly decreased MDH2 protein expression (*p* < 0.05) and the FG 0.16 mg/kg group showed significantly increased ASPH protein expression (*p* < 0.05). Fuziline and GA further regulated the expression of CASQ2, MEF2C, and ASPH in the Ca^2+^ signaling pathway by regulating the expression of MDH2 and CALR to achieve a synergistic protective effect.

## 4 Discussion

MDH2, an oxidoreductase present in the mitochondria, participates in many physiological activities, such as mitochondrial energy metabolism and active oxygen metabolism (He et al., 2022). Silencing of MDH2 reduces the intracellular ATP levels and increases the levels of ADP/ATP, NAD^+^/NADH, and ROS, which are closely related to Ca^2+^ metabolism (Ruiz et al., 2008; Xu et al., 2016). Ca^2+^ uptake in the mitochondria is necessary for ATP supply, demand balance, and energy metabolism. During myocardial cell coupling, Ca^2+^ flows into the sarcoplasmic reticulum (SR) through an L-type Ca^2+^ channel, triggering the release of Ca^2+^ in the SR, which combines with troponin C to induce myocardial cell contraction. In diastole, Ca^2+^ flows into the SR via the Ca^2+^-ATPase (SERCA) of the SR or is transported to the outside of the membrane via the Na^+^/Ca^2+^ exchanger. The activation of β_2_-AR can improve the rate and amplitude of Ca^2+^ transients in the cytosol, thereby, increasing the force on myofilaments (Hool et al., 2005). With increase in this function, the ADP hydrolyzed from ATP enters the mitochondria via the adenine nucleotide transporter, F1Fo ATPase, which is activated to regenerate ATP. This accelerates the electron flux on the electron transport chain (ETC.) and promotes the oxidation of NADH to NAD^+^. Simultaneously, Ca^2+^ enters the mitochondria via the mitochondrial calcium uniporter and activates dehydrogenases in the tricarboxylic acid cycle, enabling NADH regeneration and ETC-induced oxidation to reach a balanced state. In systolic HF, dysfunction is caused by a transient decrease in Ca^2+^ levels during the systolic period, mainly due to a decrease in the Ca^2+^ load in the SR caused by a reduction in SERCA activity and leakage of the RyR2 receptor. NADH and NADPH are oxidized in damaged myocardial cells, which reduces the ATP reduction equivalent produced by the, ETC., and triggers ROS release (Kohlhaas et al., 2017; Kohlhaas and Maack, 2010).

As the primary calcium-binding molecular protein in the endoplasmic reticulum, CALR participates in regulating calcium homeostasis, apoptosis, cardiovascular inflammation, and other physiological and pathological functions. At rest, the CALR C-domain binds to Ca^2+^, promoting Ca^2+^ storage in the endoplasmic reticulum cavity. CALR can bind to the C-terminalus of SERCA2b when glycosylated. When the Ca^2+^ concentration in the endoplasmic reticulum decreases, the two dissociate and SERCA is activated, promoting the transport of Ca^2+^ from the cytoplasm to the endoplasmic reticulum. When the Ca^2+^ concentration in the endoplasmic reticulum reaches a certain level, the two combine to inhibit the activity of SERCA, thereby reducing Ca^2+^ transport from the cytoplasm to the endoplasmic reticulum, indicating that CALR is a Ca^2+^ receptor of SERCA2b in the endoplasmic reticulum (John et al., 1998). Ischemic reperfusion causes CALR overexpression, leading to severe endoplasmic reticulum stress and Ca^2+^ homeostasis disorder, thereby activating the apoptotic signaling pathway of the endoplasmic reticulum and inducing myocardial cell damage (Lynch et al., 2006; Venkatesan et al., 2021). We verified the two target proteins using biochemical techniques and found that Fuziline and GA have a strong competitive binding relationship with MDH2 at the same concentration, suggesting that MDH2 may be the direct target of Fuziline, whereas CALR may be the specific target of GA in regulating Ca^2+^ homeostasis.

According to the results of IPA, MDH2 interacts with CALR to regulate proteins in the Ca^2+^ channel, such as CASQ2, JCN, and MEF2C. CASQ2 is the primary Ca^2+^ storage protein in the heart. As a Ca^2+^ buffer in the SR, it not only determines the Ca^2+^ storage capacity of the SR, but also participates in the release of Ca^2+^ from the SR during excitation-contraction coupling (Knollmann et al., 2006). In the presence of Ca^2+^, CASQ2 combines with the SR transmembrane protein, TRDN and JCN to form the RyR2 channel-regulating complex. During calcium release induced by cardiac excitation-contraction coupled with Ca^2+^, the Ca^2+^ level in the SR decreases and the CASQ2-T-J complex inhibits the activity of the RyR2 channel (Terentyev et al., 2008). CALR expression and RyR2 phosphorylation are promoted if CASQ2 expression i inhibited, which decreases the Ca^2+^ content in the SR and delays in Ca^2+^ reuptake (Song et al., 2007). JCN acts as a connector between CASQ2 and RyR2 (Li et al., 2015). The removal of JCN or CASQ2 from RyR2 increases the sensitivity of RyR2 to intracavitary Ca^2+^ changes, leading to the occurrence of arrhythmias induced by delayed depolarization (Lynch et al., 2005). MEF2C is a core transcription factor critical for heart development and remodeling (Yuan et al., 2018). CALR, the target of transcriptional activation of MEF2C during myocardial development, plays a key role in the nuclear translocation of MEF2C. As the intracellular Ca^2+^ concentration increases, MEF2C is dephosphorylated and transferred from the cytoplasm to the nucleus where it promotes the expression of CALR (Yuan et al., 2018). Western blotting analysis showed that Fuziline inhibited the expression of MDH2 and CALR in mice with HF and regulated the expression of MEF2C, JCN, and CASQ2 in the Ca^2+^ channel.

In this study, the optimal ratio of Fuzi and Gancao was determined, which underscores the efficacy and synergy of TCM in treating cardiomyopathy. In addition, we identified the target of the Fuzi-Gancao combination in treating cardiomyopathy and further elucidated the therapeutic mechanism. Based on our study, we can investigate more components of drug pairs and provide references for new drug development.

However, the effects of these drugs are usually systemic. The toxicity of Fuzi affects the nervous, digestive, and reproductive systems (He et al., 2023). In future, we will investigate whether Gancao, in combination with Fuzi, can be used for the treating new diseases and whether it can reduce the toxic effects of Fuzi on other organs, providing a guideline for the safe clinical use of the drug.

## 5 Conclusion

In this study, the optimum pharmacodynamic effects of Fuziline and GA were observed at compatibility ratios of 1:1 or 2:1. MDH2, which regulates the protein levels of the downstream players of the Ca^2+^ signaling pathway via CALR to balance Ca^2+^ homeostasis in the myocardial tissue and mitigate HF, is the direct target of Fuziline and GA.

## Data Availability

The original contributions presented in the study are included in the article/supplementary material, further inquiries can be directed to the corresponding author.

## Abbreviations

GA, Glycyrrhetinic acid; AHF, Acute Heart Failure; CK, Creatine Kinase; CAN, Calcineurin; cTnT, Cardiac Troponin; LDH, Lactate Dehydrogenase; MDH2, Malate Dehydrogenase 2; MEF2C, Myocyte Enhancer Factor 2C; CASQ2, Calsequestrin 2; CALR, Calreticulin; ASPH, Aspartate Beta-Hydroxylase; ROS, Reactive Oxygen Species; LVEF, Left Ventricular Ejection Fractions; LVFS, Left Ventricular Fractional Shortening; CO, Cardiac Output; LVID, left ventricular systolic inner diameter; LV VOL, left ventricular systolic volume.
